# Impacts of ovarian preservation on the prognosis of neuroendocrine cervical carcinoma: a retrospective analysis based on machine learning

**DOI:** 10.1186/s12957-023-03014-9

**Published:** 2023-05-12

**Authors:** Xuesong Xiang, Yunqiang Zhang, Keqin Hua, Jingxin Ding

**Affiliations:** 1 Department of Gynecology, The Obstetrics and Gynecology Hospital of Fudan University, 128 Shen-Yang Road, Shanghai, 200011 People’s Republic of China; 2 Shanghai Key Laboratory of Female Reproductive Endocrine-Related Diseases, The Obstetrics and Gynecology Hospital, Fudan University, Shanghai, 200011 People’s Republic of China

**Keywords:** Neuroendocrine cervical carcinoma, Ovarian preservation, Machine learning, Ovarian metastasis, Safety evaluation, Subgroup analysis

## Abstract

**Background:**

Neuroendocrine cervical carcinoma (NECC) is a rare but aggressive malignancy with younger patients compared to other common histology types. This study aimed to evaluate the impacts of ovarian preservation (OP) on the prognosis of NECC through machine learning.

**Methods:**

Between 2013 and 2021, 116 NECC patients with a median age of 46 years received OP or bilateral salpingo-oophorectomy (BSO) and were enrolled in a retrospective analysis with a median follow-up of 41 months. The prognosis was estimated using Kaplan–Meier analysis. Random forest, LASSO, stepwise, and optimum subset prognostic models were constructed in training cohort (randomly selected 70 patients) and tested in 46 patients through receiver operator curves. Risk factors for ovarian metastasis were identified through univariate and multivariate regression analyses. All data processing was carried out in R 4.2.0 software.

**Results:**

Among 116 patients, 30 (25.9%) received OP and showed no significantly different OS compared with BSO group (*p* = 0.072) and got better DFS (*p* = 0.038). After construction of machine learning models, the safety of OP was validated in lower prognostic risk group (*p* > 0.05). In patients ≤ 46 years, no impacts of OP were shown for DFS (*p* = 0.58) or OS (*p* = 0.67), and OP had no impact on DFS in different relapse risk population (*p* > 0.05). In BSO group, regression analyses showed that later stage, para-aortic LNM, and parametrial involvement were associated with ovarian metastasis (*p* < 0.05).

**Conclusions:**

Preserving ovaries had no significant impact on prognosis in patients with NECC. OP should be considered cautiously in patients with ovarian metastasis risk factors.

**Supplementary Information:**

The online version contains supplementary material available at 10.1186/s12957-023-03014-9.

## Background

Neuroendocrine cervical carcinoma (NECC) is a rare entity of gynecologic malignancy that accounts for only 1 to 3% of all cervical cancer, which are classified as small-cell type, large-cell type, and non-neuroendocrine admixed with neuroendocrine carcinoma (NEC) type [1–4]. Nevertheless, NECC is exceedingly aggressive, and cases of demise have been reported even in early stage due to locoregional recurrence or distant metastasis [5–7]. Furthermore, it was reported that NECC affected a wide age range (21–87 years) with a median age at diagnosis from 37 to 49 years old [1, 8, 9], which tends to be younger when compared with other common histology [10]. Due to the contradiction between the need for more aggressive treatment and the strong desire for ovarian retention in young women with NECC, a debate has arisen concerning criteria and rationality for ovarian preservation (OP).

Several gynecologic oncologists have suggested that OP should not be recommended in NECC. It was reported only 65% of patients who accepted OP would maintain ovarian function after external beam pelvic radiation and/or brachytherapy [11, 12], not to mention there are theoretical concerns regarding residual microscopic disease in the ovaries [13]. However, there is an alternative point of view, though it would be better to preserve one or both ovaries at the time of radical surgery in a specific group of patients for their physiological and psychosexual well-being. While bilateral salpingo-oophorectomy (BSO) eliminated microscopic carcinoma in ovaries, this procedure causes climacteric symptoms due to the abrupt loss of estrogen, including hot flushes, neurasthenia, osteoporosis, and hypertension [14–16]. Besides, increased risks of cardiovascular disease, venous thromboembolism, and breast cancer remain controversial in women undergoing oral hormone therapy [17–19]. It is also difficult to determine the suitable dosage and frequency of the drug compared with the hormone produced by the body itself [20]. Therefore, OP should be considered for partial patients whose overall health benefit would exceed the risk. However, it remains a challenge in clinical practice to discriminate a group of NECC patients that were relatively feasible for retaining ovaries.

To evaluate the safety of OP in women with NECC, the oncological outcomes were compared between women with OP and BSO in this study. The risk factors were evaluated based on machine learning, and a risk score was constructed to classify patients into subgroups, in which the impact of OP on prognosis was further discussed. Additionally, the risk factors of ovarian metastasis among women who accepted BSO were analyzed in this study.

## Materials and methods

### Patients and data collection

In approval of the Institutional Ethics Review Board of Obstetrics and Gynecology Hospital, Fudan University, a total of 10,225 patients with uterine cervical carcinoma who underwent surgical treatment from December 2013 to December 2021 were retrospectively reviewed. Of them, patients who met the following criteria were included in this study: (i) the diagnosis included pure NECC or NEC admixed with other carcinomas and (ii) underwent surgical treatment in our hospital. We excluded patients who met any one of the following conditions: (i) incomplete clinical dataset and patients who refused surgery, (ii) diagnosed as typical or atypical carcinoid tumor, (iii) distant metastasis at the first visit and diagnosed as stage 4B based on the new International Federation of Gynecology and Obstetrics (FIGO 2018) staging system, and (iv) combined with other primary cancers of non-cervical origin. All patients or their relatives signed informed consent. Of all 116 patients enrolled, 86 women underwent radical hysterectomy, pelvic lymphadenectomy, and BSO, while the remaining 30 women underwent radical hysterectomy, pelvic lymphadenectomy, and OP. To evaluate the safety of OP in a mimic clinic situation, the 116 women were randomly divided into training, and testing cohort (included 70 and 46 patients respectively) used “caret” R package.

### Pathological diagnosis

Based on the criteria of central pathological review (CPR), the diagnoses of all patients with NECC were confirmed by histologic morphology and immunohistochemical staining of tissue samples that were read by two pathologists specialized in gynecological cancers. Specifically, the pathology committee had a consensus on the diagnosis of NECC according to WHO classifications. Small-cell type was composed of hyperchromatic nuclei and scanty cytoplasm; its nuclear molding and crushing artifact were also common. Large-cell type is recognized by its arrangement in well-demarcated nests, trabeculae, or cords with peripheral palisading, and tumor cells are large and polygonal, with vesicular or hyperchromatic nuclei and a prominent nucleolus [3]. For cases with squamous differentiation or adenocarcinoma differentiation in the tumor, as long as the NEC component accounted for at least 20% of the tumor area, they were all designated as mixed histology types. Which kind of histology subtype NECC admixed with, and whether NECC was dominant in the whole tumor, is two indexes considered in the mixed histology subgroup. Furthermore, at least one of the biomarkers derived from the immunohistochemical staining, including neuron-specific enolase, chromogranin, synaptophysin, and neural cell adhesion molecule CD56, is positive. Nevertheless, positive neuroendocrine markers were not necessary for diagnosis.

### Patient, tumor, and treatment variables

The clinicopathological variables of each woman were obtained from medical records, including admission and discharge notes, as well as pathological slides. Tumor size was determined as the maximum diameter of gross tumors from pathology reports. Preoperative diagnosis was based on the pathological results of colposcopy biopsy or loop electrosurgical excision procedure (LEEP). Ovarian metastasis was defined as the occurrence of viable tumor cells in the ovarian tissues or vessels and imitated the particular cell arrangement and morphology of primary cervical neoplasm [14].

The variables analyzed included age at diagnosis, chief complaint, preoperative diagnosis, preoperative human papillomavirus (HPV) infection status (Roche cobas 4800 HPV system [Roche Molecular Systems Inc., Pleasanton, CA, USA]), tumor size, FIGO stage, histological heterogeneity (pure NECC or mixed histology types), lymph node metastasis (LNM), depth of myometrial invasion (DIM), lymph-vascular space invasion (LVSI), parametrial invasion, vaginal invasion, incisal margin involvement, lower uterine segment involvement (LUSI), and postoperative radiotherapy (chemotherapy was routinely applied to all patients and was not needed to be compared. The role of radiotherapy was controversial, for distant metastasis is much more common than local recurrence, and several studies were against that radiotherapy would promote the prognosis of NECC [21–24]). The para-aortic LNM and the LNM positive ratio were viewed as two indexes in LNM positive population.

### Statistical analysis

The primary endpoint was any NECC-related death, and the secondary one was NECC recurrence. Overall survival (OS) was measured from the date of radical hysterectomy to death or censored at the last follow-up. Disease-free survival (DFS) was measured from the date of radical hysterectomy to cancer recurrence or censored at the last follow-up. Descriptive statistics for continuous covariates are classified into higher or lower groups according to the cutoff determined by maximally selected log-rank statistics (used R package “survminer”). Categorical variables were compared through the chi-squared test and Fisher’s exact tests in our baseline table using the R package “tableone.”

Kaplan–Meier (KM) method (log-rank tests) was used to evaluate the impact of OP on prognosis. The possible significant factors that affected final risk scores (*p* < 0.1) were screened out using KM and univariate Cox regression analyses (R package survival, survminer, and ggplot2 were used). Different ways were used to construct risk scores respectively, including least absolute shrinkage and selection operator (LASSO) regression, stepwise multivariate Cox analysis, optimum subsets logistic regression, and random survival forest analysis, of which the method owned the highest area under receiver operator curve (AUC of ROC) was selected to discriminate death and relapse risks comprehensively. The safety of OP was evaluated in low- or high-risk subgroups. The R package “glmnet,” “caret,” “randomForest,” “My.stepwise,” “forestploter,” “forestplot,””bestglm,” “leaps,” “genefilter,” “Hmisc,” “ISLR,” “rms,” “regplot,” and “ROCR” were used. All data processing was carried out using R 4.2.0 software.

## Results

### The clinical and pathological features of NECC patients

**Fig. 1:**
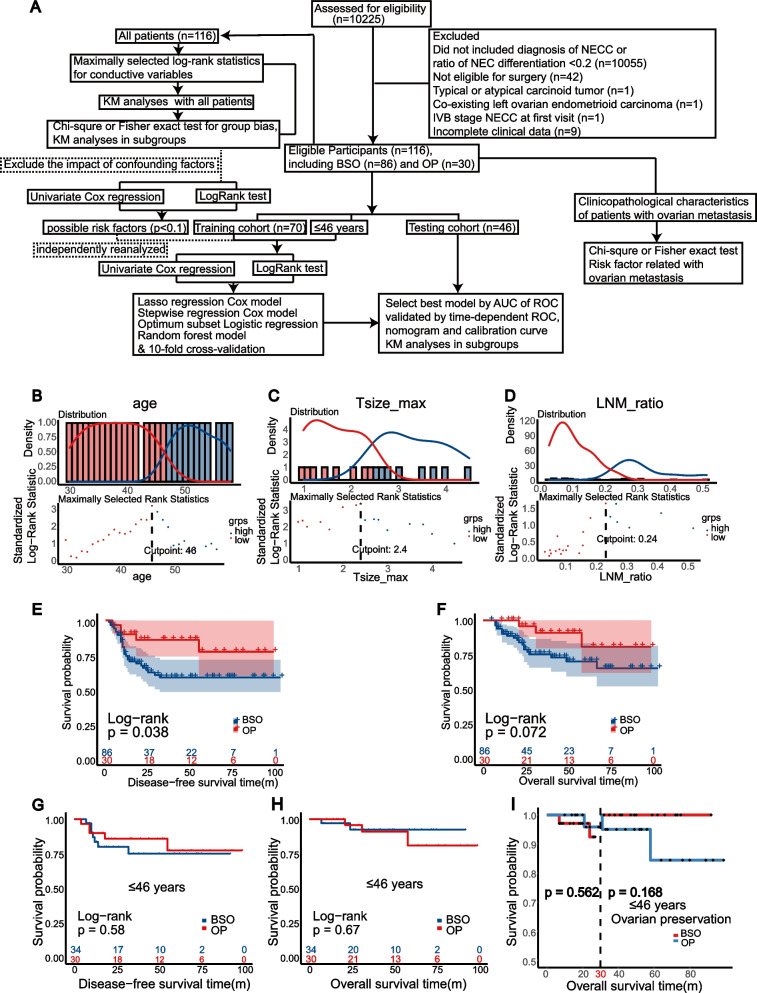
Profile of the study, the selection of optimal cut-off points, and Kaplan–Meier (KM) curves of DFS and OS between ovarian preservation (OP) and bilateral salpingo-oophorectomy (BSO) groups. **A** Flowchart of the study analysis process. **B**-**D** The optimal cut-off points to dichotomize age, tumor maximal diameter, and LNM ratio into different groups are 46 years old, 2.4 cm, and 24% (determined by survminer R package using logrank test). **E**-**F** Comparison of OP and BSO in all patients. **G**-**H** Comparison of OP and BSO in patients ≤ 46 years old. I Landmark analysis of follow-up 30 months in patients ≤ 46 years old

Until December 2021, we enrolled a total of 116 patients, 71 (61.2%) were classified as early stage, while 45 (38.8%) were locally advanced based on FIGO 2018. Figure 1A depicts the analysis workflow of this study. The median age of patients was 46 years, ranging from 22 to 76 years. The breakdown of histologic types and clinical stages of these patients was as follows: 47 (40.5%) were pure NECC, 69 (59.5%) were NECC admixed with other carcinoma, 71 (61.2%) stage 1, 13 (11.2%) stage 2, 30 (25.9%) stage 3, and 2 (1.7%) stage 4; specific distribution was shown in Supplementary Fig. 1A. They were identified with initial symptoms mainly (71.6%), while 32 cases were occasionally diagnosed on physical examination (28.4%); besides, only 56 (47.4%) patients were preoperatively diagnosed (Supplementary Fig. 1B–C). With the cutoff value of consecutive variables determined by survminer R package (46 years old, tumor maximal diameter 2.4 cm, and LNM ratio 24%), patients were characterized as younger and older groups, smaller and larger tumor groups, and lower and higher LNM ratio groups, respectively (Fig. 1B–D).

Of all 116 women, 30 had OP, while 86 accepted BSO; the clinicopathological characteristics of the patients are listed in Table 1. The median follow-up time was 41 (4–103) months, 36 (31.0%) women relapsed, and 23 (19.8%) cases of cervical cancer-related death were found in this process. There were 5 cases of recurrence in women with OP, including 4 liver and lung metastasis and 1 bone metastasis; none of them showed evidence of pelvic recurrences or ovarian relapse.

**Table 1:** Characteristics between women with ovarian preservation and bilateral salpingo-oophorectomy

Variable	*n* = 116	Ovary saved		
		**BSO**	**OP**	***P***
		**86**	**30**	
**Age (%)**				< 0.001
≤ 46	64 (55.2)	34 (39.5)	30 (100.0)	
> 46	52 (44.8)	52 (60.5)	0 (0.0)	
**FIGO stage (%)**				0.227
I	71 (61.2)	48 (55.8)	23 (76.7)	
II	13 (11.2)	11 (12.8)	2 (6.7)	
III	30 (25.9)	25 (29.1)	5 (16.7)	
IV	2 (1.7)	2 (2.3)	0 (0.0)	
**Tumor size (%)**				0.012
≤ 2.4 cm	34 (32.7)	19 (25.0)	15 (53.6)	
> 2.4 cm	70 (67.3)	57 (75.0)	13 (46.4)	
**Preoperative HPV (%)**				1
Negative	7 (8.9)	5 (8.1)	2 (11.8)	
Positive	72 (91.1)	57 (91.9)	15 (88.2)	
** Positive**				
Unknown specific type	13 (18.1)	10 (17.5)	3 (20.0)	
Known				
With HPV16	14 (23.7)	11 (23.4)	3 (25.0)	
With HPV18	46 (78.0)	37 (78.7)	9 (75.0)	
Other 12 high-risk HPVs	10 (16.9)	8 (17.0)	2 (16.7)	
**Histological heterogeneity (%)**				0.777
Pure	47 (40.5)	36 (41.9)	11 (36.7)	
Mix	69 (59.5)	50 (58.1)	19 (63.3)	
**Mix**	69			0.395
NEC differentiation	12 (17.4)	7 (14.0)	5 (26.3)	
NECC dominant	57 (82.6)	43 (86.0)	14 (73.7)	
**Mix**	69			0.3
Squamous	12 (17.4)	9 (18.0)	3 (15.8)	
Adenocarcinoma	54 (78.3)	40 (80.0)	14 (73.7)	
Both	3 (4.3)	1 (2.0)	2 (10.5)	
**LNM (%)**				0.4
Negative	84 (72.4)	60 (69.8)	24 (80.0)	
Positive	32 (27.6)	26 (30.2)	6 (20.0)	
**Positive**	32			0.732
Pelvic	28 (87.5)	22 (84.6)	6 (100.0)	
Pelvic & para-aortic	4 (12.5)	4 (15.4)	0 (0.0)	
**Positive**	32			0.136
LNM ratio low	21 (65.6)	15 (57.7)	6 (100.0)	
LNM ratio high	11 (34.4)	11 (42.3)	0 (0.0)	
**Parametrial involvement (%)**				0.09
Negative	105 (90.5)	75 (87.2)	30 (100.0)	
Positive	11 (9.5)	11 (12.8)	0 (0.0)	
**Vaginal invasion (%)**				0.02
Negative	91 (79.8)	63 (74.1)	28 (96.6)	
Positive	23 (20.2)	22 (25.9)	1 (3.4)	
**Incisal margin (%)**				0.251
Negative	107 (93.9)	78 (91.8)	29 (100.0)	
Positive	7 (6.1)	7 (8.2)	0 (0.0)	
**LUSI (%)**				0.084
Negative	94 (81.0)	66 (76.7)	28 (93.3)	
Positive	22 (19.0)	20 (23.3)	2 (6.7)	
**DIM (%)**				0.044
Superficial 1/3	32 (28.1)	20 (23.8)	12 (40.0)	
Middle 1/3	38 (33.3)	26 (31.0)	12 (40.0)	
Deep 1/3	44 (38.6)	38 (45.2)	6 (20.0)	
**LVSI (%)**				1
Negative	17 (15.6)	13 (15.9)	4 (14.8)	
Positive	92 (84.4)	69 (84.1)	23 (85.2)	
**Radiotherapy (%)**				0.229
Unaccepted	18 (18.0)	11 (14.7)	7 (28.0)	
Accepted	82 (82.0)	64 (85.3)	18 (72.0)	

*Abbreviations*: *BSO* Bilateral salpingo-oophorectomy, *OP* Ovarian preservation, *FIGO* International Federation of Gynecology and Obstetrics, *HPV* Human papillomavirus, *NEC* Neuroendocrine carcinoma, *NECC* High-grade neuroendocrine cervical carcinoma, *LNM* Lymph node metastasis, *LUSI* Lower uterine segment involvement, *DIM* Depth of myometrial invasion, *LVSI* Lymph-vascular space invasion

### Comparison of survival between NECC patients with BSO and OP

In KM analysis, it was found that OP group showed better DFS (*p* = 0.038), but this difference was not significant in OS (*p* = 0.072) (Fig. 1E–F). While it was unlikely that BSO could make the prognosis of NECC worse during our follow-up of 4–103 months, further analyses in subgroup reminded the existence of confounding factors (Table 1). Compared with BSO group, OP group owned more younger women (*p* < 0.001), more patients with smaller tumor diameter (*p* = 0.012), more negative vaginal invasion (*p* = 0.02), and more superficial myometrial invasion (*p* = 0.044) based on pathological report. Other clinicopathological factors were uniform in these 2 groups.

It is undoubtedly that the dilemma of OP mainly happened in younger women, which also showed by no women > 46 years chose to preserve their ovaries in our study. Therefore, younger patients (≤ 46 years old) were analyzed separately. After this, the population characteristics were mostly uniform (Supplementary Table 1) except for tumor diameter (*p* = 0.044). The survival curves between BSO and OP group showed similar DFS (*p* = 0.58) and OS (*p* = 0.67) (Fig. 1G–H). However, there was an intersection between BSO and OP groups in OS curves when followed up about 30 months, and OP group possibly showed a worse long-term prognosis. Therefore, we used 30 months as a landmark to further analyzed the curves, which showed no significant difference (*p* = 0.168 after 30 months, Fig. 1I).

Furthermore, we compared OS and DFS between BSO and OP groups in the three different DIM and vaginal invasion statuses based on data from all patients and different tumor diameter based on all and younger patients. All of them showed no significant difference (*p* < 0.05, Supplementary Fig. 1D–K). However, it should be noted that only one patient with positive vaginal invasion accepted OP and died of recurrence after 58 months of following up (but not reported ovarian metastasis).

### Risk factors significantly affecting the prognosis of NECC patients

**Fig. 2:**
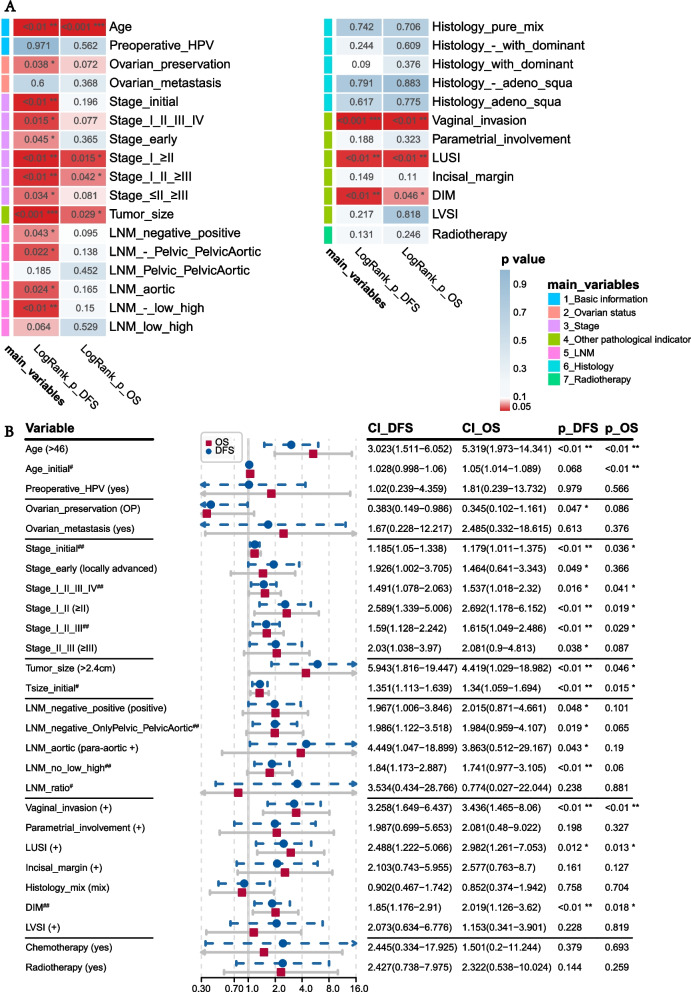
KM and univariate Cox analyses of the impacts of clinicopathological variables on prognosis. **A** The heatmap showed the logrank test *p*-value of 7 important clinicopathological variable groups for DFS and OS. The color changes from blue to red when *p*-value decreases. The pheatmap R package was used to draw the heatmap. The names of variables indicate the method of grouping as follows: “LNM_-_Pelvic_PelvicAortic” represents classifying as LNM negative, only pelvic LNM and pelvic and para-aortic LNM; “Histology_-_with_dominant” represents classifying as pure NECC, with NEC differentiation, and NECC is dominant in histology. **B** Univariate Cox regression of all important clinicopathological variables for DFS and OS, hazard ratio, and 95% confidence interval was shown in forest plot using the “forestplot” R package. #Import into univariate Cox model as consecutive variable originally. ##Import into univariate Cox model as consecutive variables by numbering, stage from early to late, LNM from negative to high, or increase from only pelvic to pelvic and para-aortic LNM, DIM from superficial to deep

Then, we screened out the important factors to construct a risk score that can predict prognosis comprehensively. All clinical and pathological factors studied and the log-rank test *p*-values were displayed through the heatmap (Fig. 2A). Among them, older patients (> 46 years old, *p* = 0.001 for OS, *p* = 0.00022 for DFS); larger tumor (> 2.4 cm, *p* = 0.00081 for DFS, *p* = 0.029 for OS); positive vaginal invasion (*p* = 0.00031 for DFS, *p* = 0.0026 for OS); lower uterine segment involved (LUSI) (*p* = 0.0091 for DFS and OS); and deeper myometrial invasion (*p* = 0.0062 for DFS, *p* = 0.046 for OS) were all significant prognostic factors related to poor DFS and OS (Supplementary Fig. 2A–J).

FIGO stage was classified as multiple variables by different divisions, with the optimum cut-off point being earlier than IB3 (Supplementary Fig. 2K–L); stage 1 and above 2 showed minimal *p*-value (*p* = 0.0033 for DFS, *p* = 0.015 for OS) (Supplementary Fig. 2M–N). Besides, classifying as earlier than 2 and above 3 was usual in clinical practice and also showed significant impact on DFS (*p* = 0.034) (Supplementary Fig. 2O–P). LNM was mainly associated with DFS (*p* = 0.043 for DFS, *p* = 0.095 for OS) (Supplementary Fig. 2Q–R); the para-aortic LNM and higher LNM ratio also significantly affected DFS (*p* < 0.05, Supplementary Fig. 2S–U).

The univariate Cox regression analysis validated older age (> 46 years), larger tumor (> 2.4 cm), later stage, vaginal invasion, LUSI, and deeper DIM were predictive risk factors for DFS and OS; para-aortic and pelvic LNM positive, or higher LNM ratio (> 24%), were risk factors for DFS (Fig. 2B).

### Evaluating the safety of OP in different prognostic groups classified by machine learning

Randomly, 70 patients were included in the training cohort to establish the model, and 46 patients were entered into the testing cohort. We used the “caret” R package to complete the grouping process, and the clinicopathological characteristics of the two cohorts were shown in Supplementary Table 2.

**Fig. 3:**
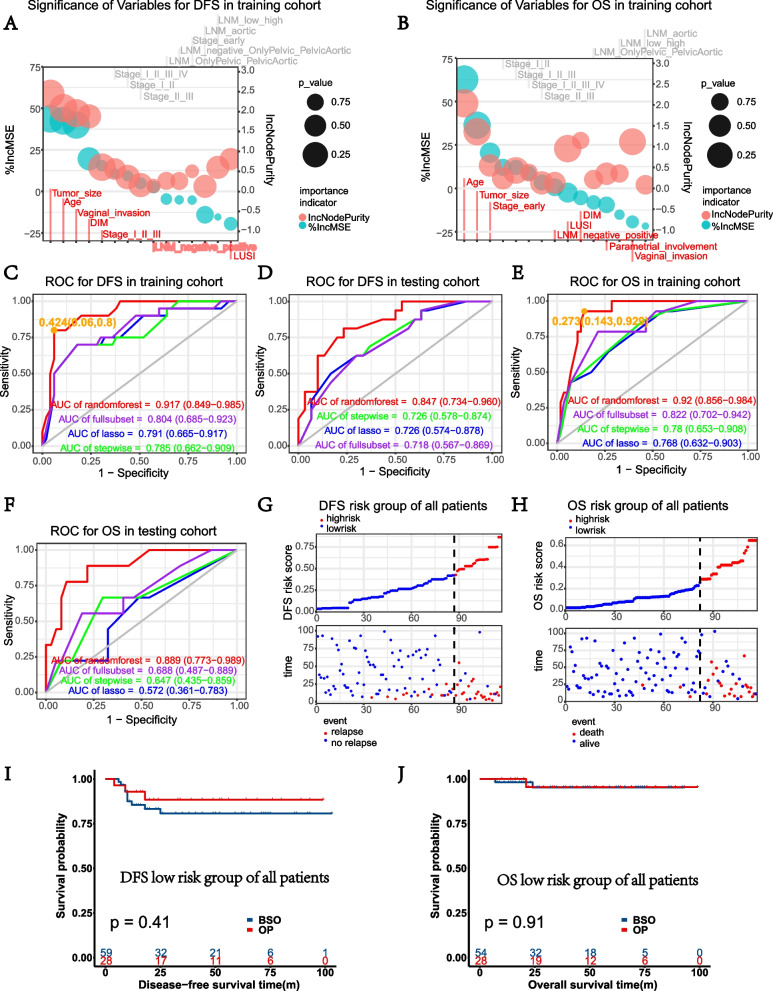
The construction of risk scores for prognosis and evaluation of OP in training and testing cohorts. **A**–**B** The significance order of variables related to DFS and OS using the random forest model in training cohort. “%IncMSE” means “increase in mean squared error (%),” and “IncNodePurity” means “increase in node purity,” both of which showed the significance of variables, choosing “%IncMSE” as the primary index. The randomForest R package was used. **C**–**D** ROC of the established models in predicting DFS of the training and testing cohort and AUC were compared. **E**–**F** ROC of the established models in predicting OS of the training and testing cohort and AUC were compared. **G**–**H** The proportion of relapse and death in high- and low-risk groups of all patients as risk scores increased. **I**–**J** KM curves of DFS and OS between BSO and OP groups in low DFS and OS risk patients respectively

The possible risk factors for DFS and OS (*p* < 0.1), which were screened out from all patients, were reanalyzed in the training cohort using KM and uni-Cox. Then, Lasso regression, optimum subset regression, and stepwise regression were performed with tenfold cross-validation in the training cohort (Supplementary Fig. 3A–H). The random forest analysis decided different significant orders of variables when considering DFS or OS respectively in training cohort (Fig. 3A–B). Tumor size, age, vaginal invasion, DIM, stage (1, 2, and ≥ 3), LNM, and LUSI were calculated to predict DFS, while age, tumor size, stage (whether in early stage), LNM, LUSI, DIM, parametrial involvement, and vaginal invasion were used to predict OS in random forest models. Then, the completed models were applied to the testing cohort. Random forest model owned maximal AUC of ROC both for relapse and death (*AUC* = 0.917 and 0.847 for DFS in training and testing cohort respectively, *AUC* = 0.920 and 0.889 for OS in training and testing cohort respectively, Fig. 3C–F).

The scatter diagram of DFS and OS risk scores showed our models discriminated the prognosis of NECC patients well (Fig. 3G–H). There were few patients in high recurrence, or death risk groups chose OP (Supplementary Table 3), and the comparison of BSO and OP in lower risk groups showed no significant impact on prognosis (*p* = 0.41 for DFS and *p* = 0.91 for OS, Fig. 3I–J). The distribution of different risk groups and KM analysis in training and testing cohort certified the credibility of our risk models; the time-dependent ROC showed the stability of the models predicted 1-, 3-, and 5-year prognoses (0.82, 0.90, 0.90 for 1-, 3-, and 5-year relapse, 0.89, 0.89, 0.92 for 1, 3, and 5 years’ death), and OP showed no significant impact on prognoses of lower risk groups whether in training or testing cohort (Supplementary Fig. 3I–X). The detailed DFS and OS risk scores and related variables were shown in Supplementary Tables 4, 5.

### Evaluating the safety of OP in different risk subgroups of younger patients less than 46 years old

**Fig. 4:**
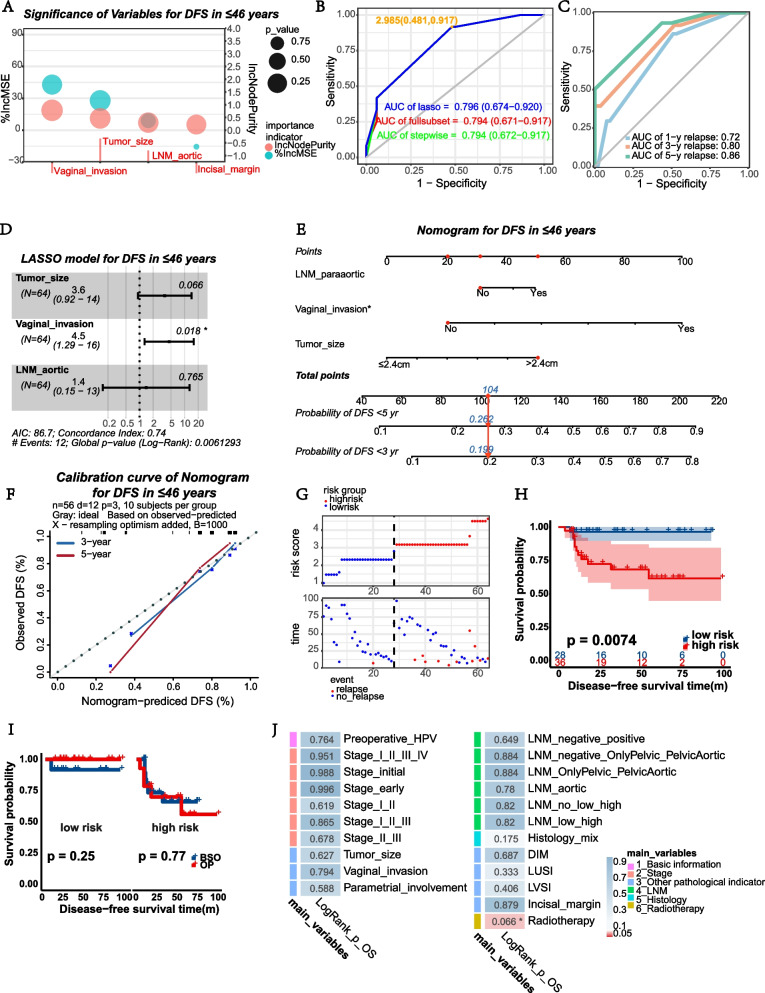
Evaluation of ovarian preservation in patients ≤ 46 years old. **A** The significance order of variables related to DFS using the random forest model in patients ≤ 46 years old. **B** ROC of the established models in predicting DFS of patients ≤ 46 years old, and AUC were compared. **C** ROC of the LASSO model for relapse at 1, 3, and 5 years in patients ≤ 46 years old. **D** The forest plot of the variables related to DFS chosen by the LASSO model in patients ≤ 46 years old. **E** Nomogram of the LASSO model for DFS in patients ≤ 46 years old. **F** Calibration curve of the nomogram used for DFS in patients ≤ 46 years old based on LASSO analysis. **G** The proportion of relapse in higher and lower risk groups of patients ≤ 46 years old as risk scores increased. **H** KM curves of DFS between lower and higher risk groups classified by LASSO analysis in patients ≤ 46 years old. **I** KM curves of DFS between BSO and OP groups in lower and higher risk groups of patients ≤ 46 years old. **J** The heatmap showed the logrank test *p*-value of 6 important clinicopathological variable groups for OS in patients ≤ 46 years old

In 64 patients who are less than 46 years old, the possible risk factors associated with DFS were reanalyzed and screened out vaginal invasion, tumor size, incisal margin, and para-aortic LNM as predictors. The random forest model gave out the significance order of the variables (Fig. 4A). After that, we constructed risk scores through LASSO, stepwise regression, and optimum subset regression analysis with tenfold cross validation (Supplementary Fig. 4A–E) and picked out the LASSO model for its highest AUC of ROC (*AUC* = 0.796 for DFS in patients younger than 46 years old, Fig. 4B). The AUC of predicted recurrence risk was stable in 1, 3, and 5 years (*AUC* = 0.72, 0.80, 0.86 for 1, 3, and 5 years, respectively, Fig. 4C). The forest plot showed the concordance index of LASSO was 0.74, and the model discriminated DFS well (global *p*-value of the model was 0.006, Fig. 4D). Therefore, the nomogram and its calibration curve were constructed based on the LASSO analysis; NECC patients less than 46 years old would be able to estimate their probability of recurrence in the future (Fig. 4E–F).

After that, the enrolled patients who are less than 46 years were divided into relatively higher risk group and lower risk group of recurrence, and different DFS were compared through KM analysis (*p* = 0.0074 for DFS between two relapse risk groups, Fig. 4G–H). Detailed risk factors and scores of each patient were shown in Supplementary Table 6, and there was essential equivalence of patients in two DFS risk group chose OP (Supplementary Table 3). OP was validated with no impact on DFS in lower and higher relapse risk populations (*p* = 0.25 for lower risk cohort, *p* = 0.77 for higher risk group, Fig. 4I). The analysis of possible risk factors related to OS through KM and uni-Cox found only radiotherapy possibly affected OS in patients ≤ 46 years (*p* = 0.066 between the OS of patients who accepted radiotherapy or not, Fig. 4J and Supplementary Fig. 4F).

### Risk factors significantly associated with ovarian metastasis

Visible or invisible ovarian metastasis is a crucial factor for the safety of OP in patients with NECC when radical surgery is performed. Of the 86 NECC patients in our study who underwent a radical hysterectomy, pelvic lymphadenectomy, and BSO, 3 (3.5%) had ovarian metastasis, and their detailed information was given (Supplementary Table 7). The univariate analysis showed that the later FIGO stage (*p* = 0.001), para-aortic LNM (*p* = 0.015), and parametrial involvement (*p* < 0.05) were associated with ovarian metastasis (Table 2). However, multivariate logistic regression analysis with possible factors (*p* < 0.1 in univariate analysis) showed that no factor was independently associated with ovarian metastasis.

**Table 2:** Incidence of ovarian metastasis according to each risk factor

Variable	Total, *n* = 86	OM, *n*	%	*P*
**Age (%)**				1
≤ 46	34	1	2.94	
> 46	52	2	3.85	
**FIGO stage (%)**				0.001
I	48	0	0.00	
II	11	1	9.09	
III	25	1	4.00	
IV	2	1	50.00	
**Tumor size (%)**				1
≤ 2.4 cm	19	0	0.00	
> 2.4 cm	57	1	1.75	
**Histological heterogeneity (%)**				0.771
Pure	36	2	5.56	
Mix	50	1	2.00	
** Mix**				1
NEC differentiation	7	0	0.00	
NECC dominant	43	1	2.33	
** Mix**				0.880
Squamous	9	0	0.00	
Adenocarcinoma	40	1	2.50	
Both	1	0	0.00	
**LNM (%)**				0.448
Negative	60	1	1.67	
Positive	26	2	7.69	
** Positive**				0.015
Pelvic	22	0	0.00	
Pelvic & para-aortic	4	2	50.00	
** Positive**				0.330
LNM ratio low	15	0	0.00	
LNM ratio high	11	2	18.18	
**Parametrial involvement (%)**				0.050
Negative	75	1	1.33	
Positive	11	2	18.18	
**Vaginal invasion (%)**				0.332
Negative	63	1	1.59	
Positive	22	2	9.09	
**Incisal margin (%)**				0.383
Negative	78	1	1.28	
Positive	7	1	14.29	
**LUSI (%)**				0.264
Negative	66	1	1.52	
Positive	20	2	10.00	
**DIM (%)**				0.588
Superficial 1/3	20	0	0.00	
Middle 1/3	26	1	3.85	
Deep 1/3	38	2	5.26	
**LVSI (%)**				1
Negative	13	0	0.00	
Positive	69	3	4.35	

*Abbreviations*: *OM* Ovarian metastasis, *FIGO* International Federation of Gynecology and Obstetrics, *NEC* Neuroendocrine carcinoma, *NECC* High-grade neuroendocrine cervical carcinoma, *LNM* Lymph node metastasis, *LUSI* Lower uterine segment involvement, *DIM* Depth of myometrial invasion, *LVSI* Lymph-vascular space invasion

## Discussion

Considering the relatively poorer prognosis of NECC in previous studies, there are no data to support the consideration of fertility preservation, such as simple conization or radical trachelectomy, even in patients with early-stage disease. While fertility-sparing surgeries have also been reported in women with early-stage NECC [25, 26], most gynecologic oncologists tend to apply more aggressive treatment, which contributed to the tendency of BSO in clinical practice. However, OP is thought to be particularly important for premenopausal women, who might be more common in NECC than other histology types [1, 8–10]. But there was no consensus on the safety of OP in NECC. In this study, it was found that OP is safe in patients with NECC, especially in younger patients who owned better prognoses based on the machine learning model.

Low incidence and lack of prospective clinical trials made it difficult to draw conclusions on the management of NECC despite the urgent need of clinical practice [21, 27, 28]. Therefore, the outcomes of women with NECC who chose OP were rarely reported. It was inferred from a study with 1965 patients that non-squamous histology should be a deterrent to OP due to the possibility of residual microscopic tumor [29], yet whether the cases of NECC are included in this study was not pointed out. Zhang et al. found that BSO may improve the prognosis of patients through the comparison of KM curves, especially for OS (*p* = 0.023). However, the impact was not significant for DFS (*p* = 0.235); besides, this conclusion was not validated in univariate and multivariate cox regression analysis of their study [30]. Furthermore, selection bias could have existed since it was obvious that several other significant risk factors, such as age, tumor size, and FIGO stage, would possibly affect patients’ choice of OP and their prognosis. In our study, the results compared KM curves of OP, and BSO in all enrolled patients showed OP had no significant effects on OS and even had a better prognosis for DFS. However, the adverse effects of BSO, such as cardiovascular disease or osteoporosis, would mostly occur in longer-term follow-up, and both death and recurrence were NECC related in our study [14–16]. Thus, the existence of confounding factors was reminded. Additionally, for the possible risk factors that caused bias between the prognosis of OP and BSO groups, such as age, tumor size, and DIM, KM analysis in subgroups respectively showed OP still did not influence prognosis significantly.

The investigation of the most significant prognostic factors could help us discriminate the risk of patients comprehensively, thus evaluating the safety of OP in different risk subgroups. From previous studies, significant prognostic variables are varied, which may include age, FIGO stage, tumor size, LNM, LVSI, DIM, histology heterogeneity, and the use of adjuvant therapies [4, 27, 31–33]. And whether the different risk population classified by these variables was safe to accept OP was uncertain. Our patients were divided into training and testing cohorts, and LASSO, stepwise, optimum subsets, and random forest models were constructed and validated through tenfold cross validation in training cohort. It was demonstrated that random forest models owned highest AUC in testing cohort whether for DFS or OS. Therefore, tumor size, age, vaginal invasion, DIM, stage (1, 2, and ≥ 3), LNM, and LUSI were calculated to predict DFS, while age, tumor size, stage (whether in early stage), LNM, LUSI, DIM, parametrial involvement, and vaginal invasion were used to estimate OS. Then, prognoses between OP and BSO were compared through KM analysis, and it was validated that OP should be considered if the patients wished in the population of lower risk. However, no patients with higher risk have chosen OP, which might be related to the age of the high-risk prognosis group (82.8% patients of high DFS risk group and 100% patients of high OS risk group were > 46 years old).

After that, OP was evaluated in the cohort of younger women (≤ 46 years old) independently. Vaginal invasion, tumor size, and para-aortic LNM were used to construct recurrence risk scores and divided patients into different groups, in which the safety of OP was confirmed. On the other hand, only accepting radiotherapy was found to be possibly associated with death risk in patients ≤ 46 years, and none of the patients in this study had pelvic recurrence. It might remind us that the risks and benefits of radiotherapy need further estimation, though theoretically radiotherapy was recommended in patients with higher risks. Besides, the impact of radiotherapy on ovarian function should be considered for women who require OP [11, 12]. In our present study, several strategies were applied to preserve ovarian function, for instance, all of the preserved ovaries were suspended outside the radiation field, and GnRH-α was used for young patients 14 days before chemotherapy. In total, for those patients ≤ 46 years, there was no significant effect of OP on prognosis even in higher risk population. Therefore, the need to preserve ovaries in these patients should be considered in treatment, with necessary strategies for protecting ovarian function.

Notably, one main concern for the safety of OP in NECC would be ovarian metastasis. The ratio of ovarian metastasis was seldomly mentioned in published studies of NECC and was higher than other common histology. A study of 133 NECC patients found 2 (1.5%) cases of ovarian metastasis at diagnosis [30], which was higher than 0.9% reported in whole cervical carcinoma [29]. Ngamcherttakul et al. even concluded that non-neuroendocrine would be the prerequisite of OP since one of two enrolled NECC patients occurred ovarian metastasis in their study [13]. In our study, 3 (3.5%) ovarian metastases were found in 86 women who underwent BSO based on the pathological reports. While the conclusion was affected by rarity, the univariate analysis showed that the incidence of ovarian metastasis was increased in patients with later FIGO stage, para-aortic LNM, and parametrial involvement (*p* < 0.05). Besides, none of the patients in subgroups of these factors (stage 4, para-aortic LNM, and parametrial involvement) accepted OP, though para-aortic LNM has been considered in our DFS risk model for patients ≤ 46 years old. Therefore, for patients who found these risk factors pre- and intraoperatively, OP should be cautiously considered, and the possibility of ovarian metastasis should be ruled out.

The main limitation of this study is that it is a retrospective study with limited sample size. Though the rarity of NECC might restrict the implementation of prospective randomized studies, large sample sized retrospective study with longer follow-up period is warranted to evaluate the safety of OP. Another limitation is that the patients enrolled were required to be eligible for surgery and had diseases in earlier stages than other studies of NECC, which is also reflected by lower rates of nodal and distant metastasis and higher 5-year overall survival rate [5, 10, 31, 33]. However, in general, our risk model would be more applicable in patients eligible for surgery to consider OP, rather than risk prediction in all populations.

## Conclusions

Preserving ovaries had no significant impact on prognosis of NECC patients. Ovarian preservation needs of women with NECC, whether in low-risk or high-risk prognosis group, should be taken into account in their therapeutic strategies. If high-risk factors associated with ovarian metastasis were identified pre- and intraoperatively, such as stage 4, para-aortic LNM, and parametrial involvement (*p* < 0.05), OP should be considered very cautiously, and more thorough preoperative evaluation and intraoperative examination should be validated to rule out the possibility of ovarian metastasis.

## Supplementary Information

**Additional file 1:**  **Supplementary Fig. 1.** (**A**-**C**) Three pie charts respectively showed the distribution of pathological stages, chief complaints, and preoperative pathological diagnoses in all 116 NECC patients. (**D**-**E**) Comparison of OP and BSO in all patients with different depths of myometrial invasion. (**F**-**G**) Comparison of OP and BSO in all patients with different tumor maximal diameters. (**H**-**I**) Comparison of KM curves between BSO and OP for DFS and OS in different vaginal invasion status groups. (**J**-**K**) Comparison of OP and BSO in patients ≤46 years old with different tumor maximal diameters

**Additional file 2:** **Supplementary Fig. 2. (A-J)** KM curves of age, tumor maximal diameter, vaginal invasion, LUSI, and DIM for DFS and OS. **(K-L)** The optimal cut-off points to dichotomize FIGO stage into earlier and later (IB3 when FIGO stage transformed into a conductive variable). **(M-R)** KM curves of the stage (I and ≥II, ≤II and ≥III) and LNM for DFS and OS. **(S-U)** KM curves of LNM subgroups (para-aortic LNM and LNM ratio) for DFS and OS.

**Additional file 3:** **Supplementary Fig. 3. (A)** LASSO coefficient profiles of the 11 key clinicopathological variables for the prediction of DFS in the training cohort. **(B)** Tuning parameter selection by tenfold cross-validation in the LASSO model of training cohort. The partial likelihood deviance was plotted against log (Lambda/λ), and λ was the tuning parameter. The partial likelihood deviance values were shown and error bars represented s.e. The dotted vertical lines showed the optimal values through minimum criteria and 1-s.e. criteria. **(C)** Tenfold cross-validation showed that 4 variables could minimize the error of optimum subsets regression analysis in the training cohort. The “bestglm” R package was used. **(D)** The Cox model display the variables related to DFS chosen by stepwise regression in the training cohort.** (E)** LASSO coefficient profiles of the 13 key clinicopathological variables for the prediction of OS in the training cohort. **(F)** Tuning parameter selection by tenfold cross-validation in the LASSO model of training cohort. **(G)** Tenfold cross-validation showed that 3 variables could minimize the error of optimum subsets regression analysis in the training cohort. **(H)** The Cox model of the variables related to OS chosen by stepwise regression in the training cohort.** (I-J)** The proportion of relapse in high and low risk groups of the training and testing cohort as risk scores increased.** (K-M)** ROC of the random forest model for relapse at 1, 3, and 5 years in the training, testing cohort, and all patients.** (N)** KM curves of DFS between lower and higher risk groups classified by random forest analysis in all patients.** (O-P)** KM curves of DFS between BSO and OP groups in low DFS risk patients of training and testing cohort.** (Q-R)** The proportion of death in high and low risk groups of the training and testing cohort as risk scores increased.** (S-U)** ROC of the random forest model for death at 1, 3, and 5 years in the training, testing cohort, and all patients.** (V)** KM curves of OS between lower and higher risk groups classified by random forest analysis in all patients.** (W-X)** KM curves of OS between BSO and OP groups in low OS risk patients of training and testing cohort.

**Additional file 4:** **Supplementary Fig. 4. (A)** LASSO coefficient profiles of the 4 key clinicopathological variables for the prediction of DFS in patients ≤46 years old. **(B)** Tuning parameter selection by tenfold cross-validation in the LASSO model of patients ≤46years old. **(C)** Tenfold cross-validation showed that 4 variables could minimize the error of optimum subsets regression analysis in patients ≤46 years old. **(D)** The Cox model of the variables related to DFS chosen by optimum subsets regression in patients ≤46 years old. **(E)** The Cox model of the variables related to DFS chosen by stepwise regression in patients ≤46 years old. **(F)** KM curves of OS between whether accepted radiotherapy in patients ≤ 46 years old.

**Additional file 5:** **Table S1.** Characteristics between women ≤46 years of age with ovarian preservation or bilateral salpingo-oophorectomy. **Table S2.** Clinicopathologic characteristics of the training and testing population. **Table S3.** The selection of bilateral salpingo-oophorectomy and ovarian preservation in the lower or higher risk of death and relapse groups.

**Additional file 6: Table S4.** Detailed DFS risk scores and related variables.

**Additional file 7: Table S5.** Detailed OS risk scores and related variables.

**Additional file 8: Table S6.** Detailed DFS risk scores and related factors of patients younger than 46 years old.

**Additional file 9:** **Table S7. **Clinical and pathological characteristics of patients with ovarian metastasis.

## Data Availability

The raw data supporting the conclusions of this article will be made available by the authors, without undue reservation.
