# Supplementary figures and images for: Impacts of ovarian preservation on the prognosis of neuroendocrine cervical carcinoma: a retrospective analysis based on machine learning
Source: World J Surg Oncol. 2023 May 12;21:146. doi: 10.1186/s12957-023-03014-9 (PMC10176922; doi:10.1186/s12957-023-03014-9)

A

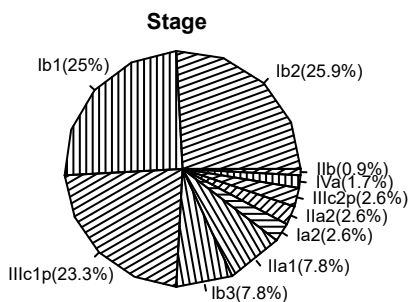

B

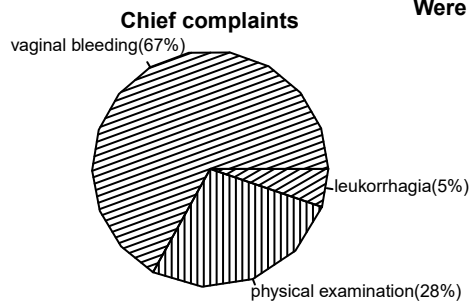

C

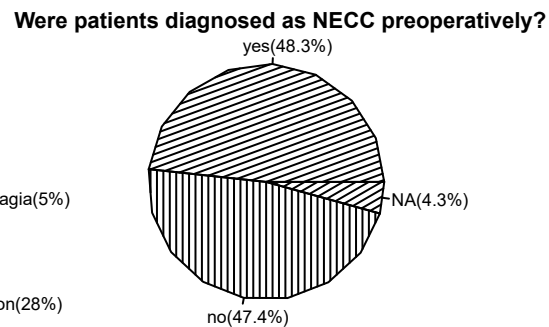

D

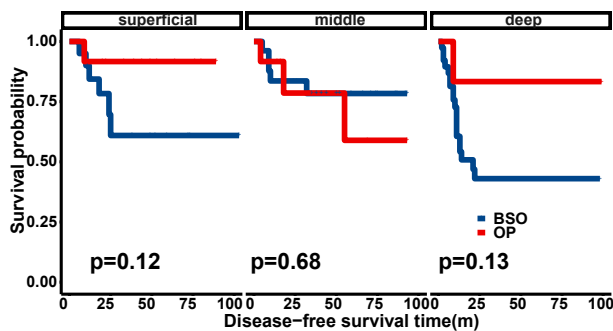

E

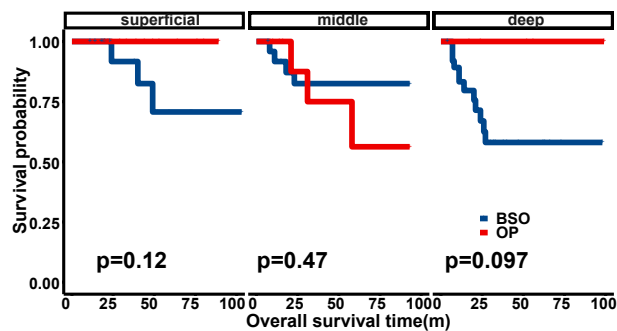

F

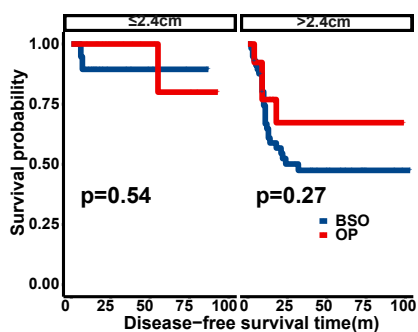

G

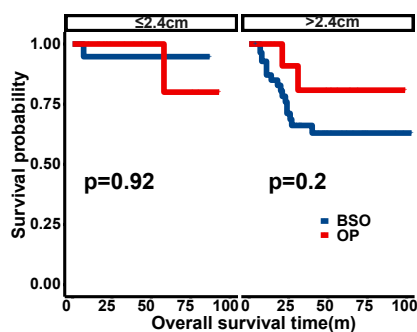

H

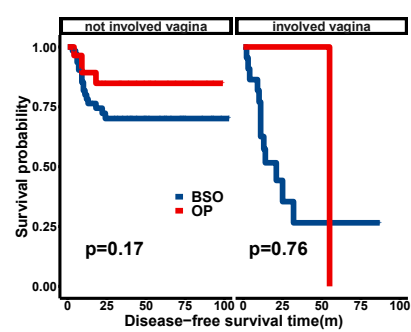

I

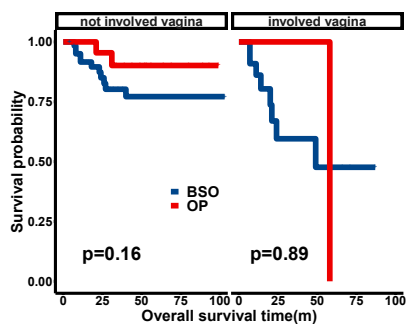

J

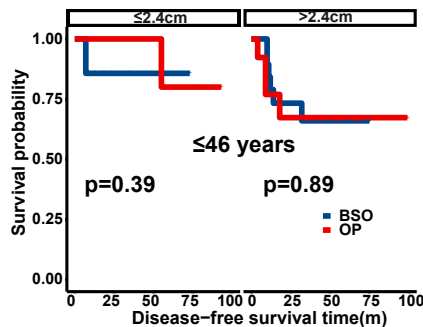

K

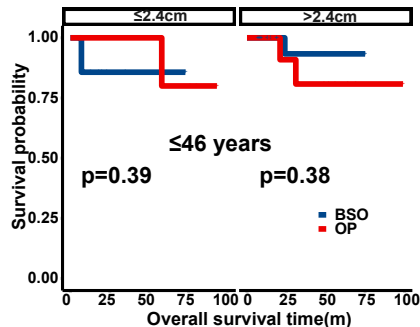

Supplement: Supplementary file 1 — Additional file 1: Supplementary Fig. 1. (A-C) Three pie charts respectively showed the distribution of pathological stages, chief complaints, and preoperative pathological diagnoses in all 116 NECC patients. (D-E) Comparison of OP and BSO in all patients with different depths of myometrial invasion. (F-G) Comparison of OP and BSO in all patients with different tumor maximal diameters. (H-I) Comparison of KM curves between BSO and OP for DFS and OS in different vaginal invasion status groups. (J-K) Comparison of OP and BSO in patients ≤46 years old with different tumor maximal diameters [file 12957_2023_3014_MOESM1_ESM.pdf]

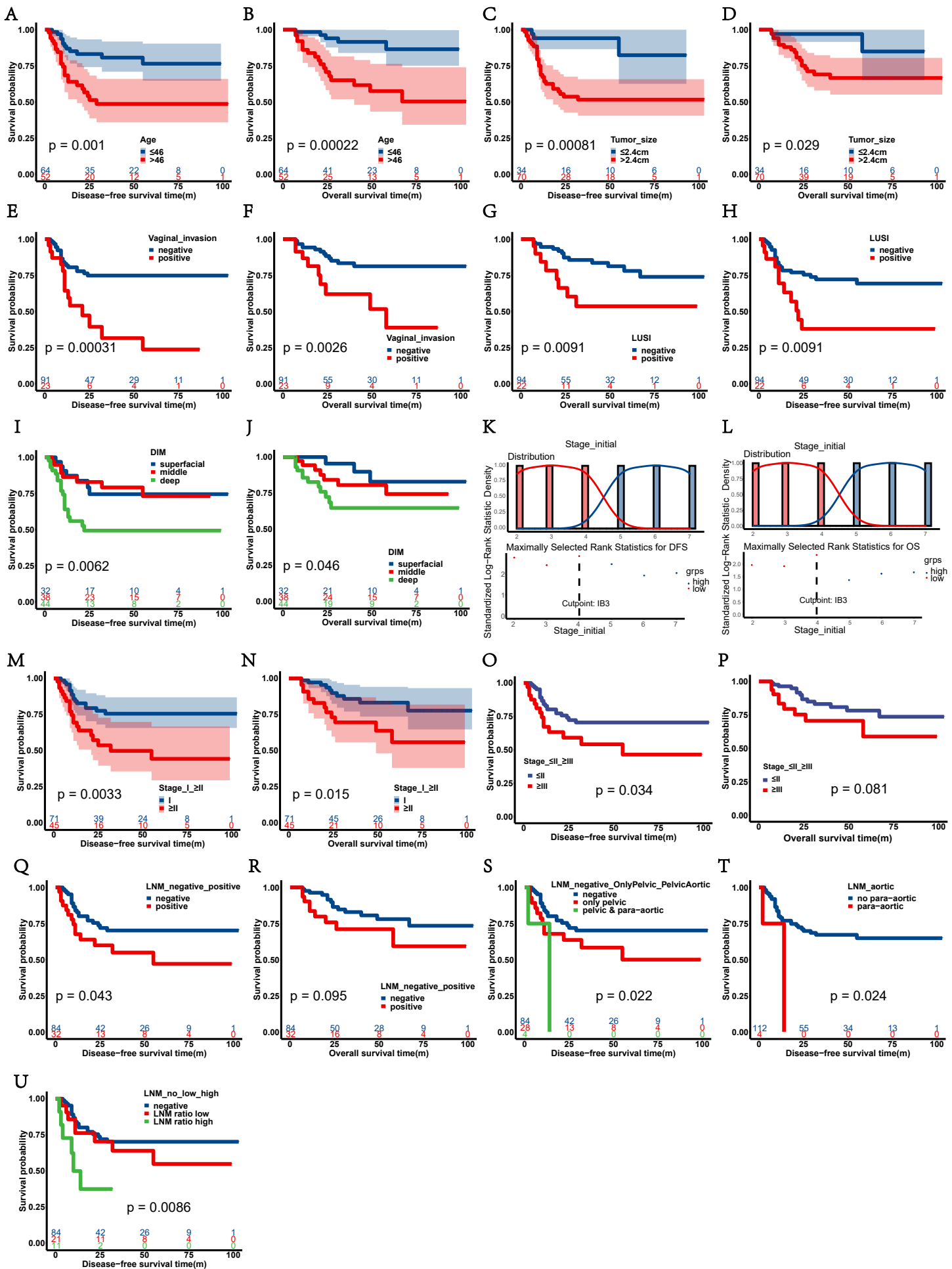

Supplement: Supplementary file 2 — Additional file 2: Supplementary Fig. 2. (A-J) KM curves of age, tumor maximal diameter, vaginal invasion, LUSI, and DIM for DFS and OS. (K-L) The optimal cut-off points to dichotomize FIGO stage into earlier and later (IB3 when FIGO stage transformed into a conductive variable). (M-R) KM curves of the stage (I and ≥II, ≤II and ≥III) and LNM for DFS and OS. (S-U) KM curves of LNM subgroups (para-aortic LNM and LNM ratio) for DFS and OS. [file 12957_2023_3014_MOESM2_ESM.pdf]

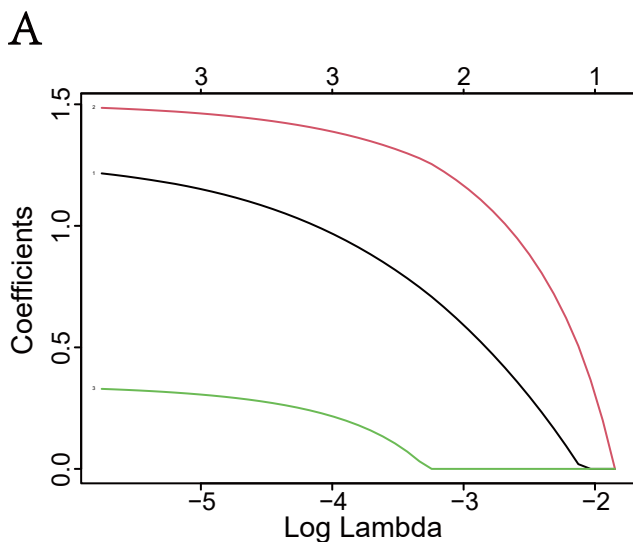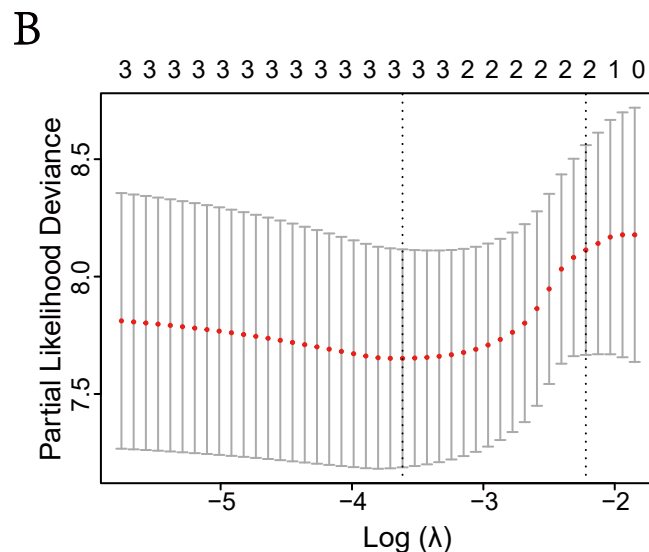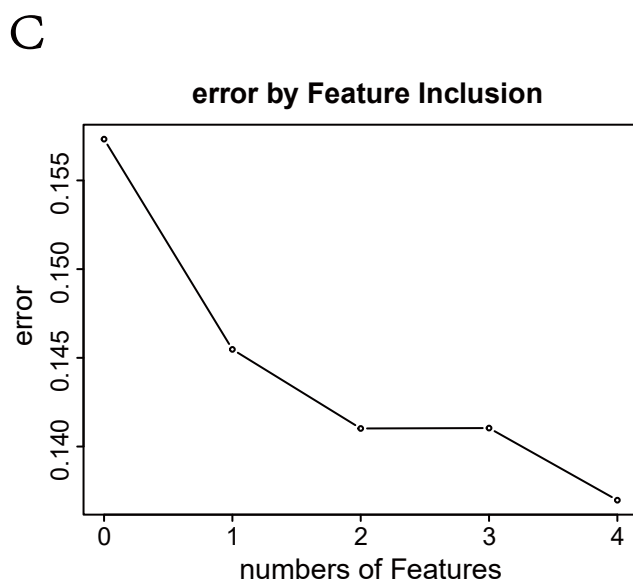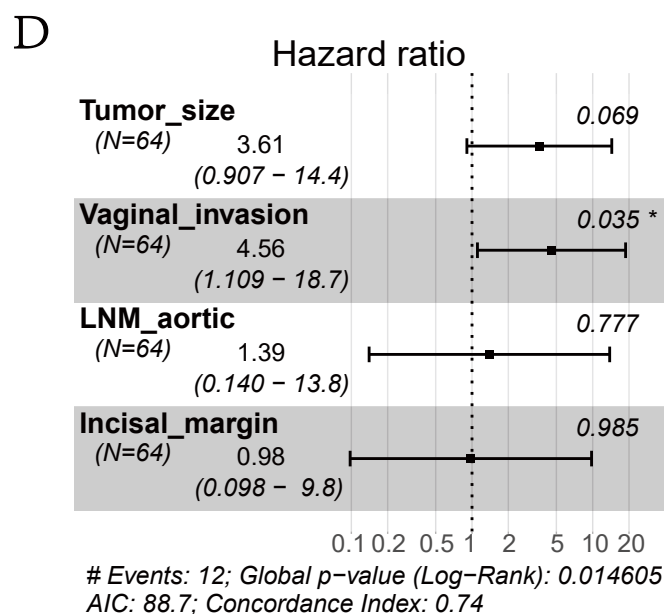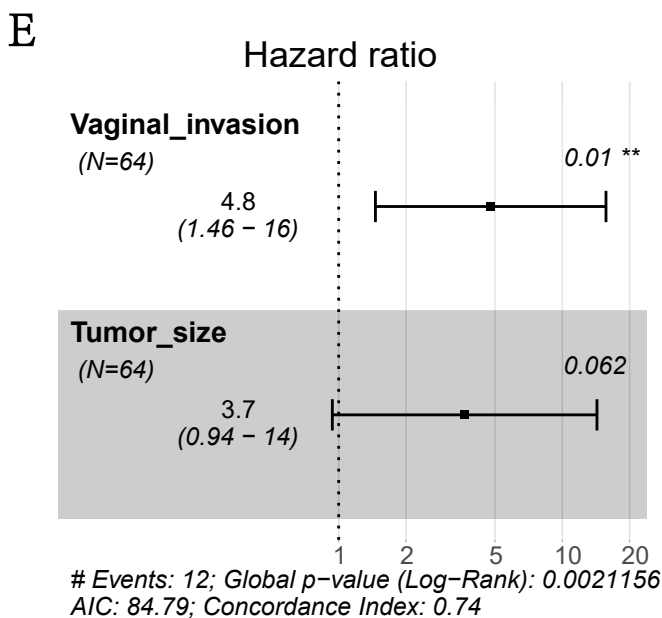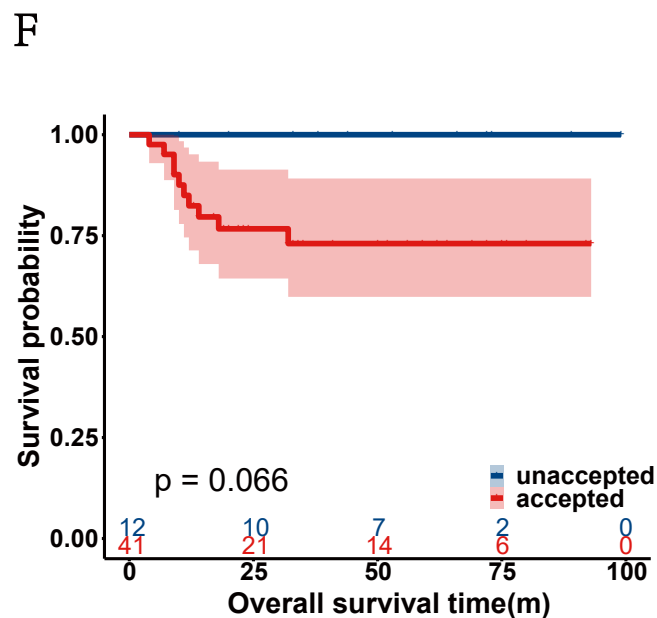

Supplement: Supplementary file 4 — Additional file 4: Supplementary Fig. 4. (A) LASSO coefficient profiles of the 4 key clinicopathological variables for the prediction of DFS in patients ≤46 years old. (B) Tuning parameter selection by tenfold cross-validation in the LASSO model of patients ≤46years old. (C) Tenfold cross-validation showed that 4 variables could minimize the error of optimum subsets regression analysis in patients ≤46 years old. (D) The Cox model of the variables related to DFS chosen by optimum subsets regression in patients ≤46 years old. (E) The Cox model of the variables related to DFS chosen by stepwise regression in patients ≤46 years old. (F) KM curves of OS between whether accepted radiotherapy in patients ≤ 46 years old. [file 12957_2023_3014_MOESM4_ESM.pdf]
