# Supplementary material for: Impacts of ovarian preservation on the prognosis of neuroendocrine cervical carcinoma: a retrospective analysis based on machine learning
Source: World J Surg Oncol. 2023 May 12;21:146. doi: 10.1186/s12957-023-03014-9 (PMC10176922; doi:10.1186/s12957-023-03014-9)
Supplement: Supplementary file 5 — Additional file 5: Table S1. Characteristics between women ≤46 years of age with ovarian preservation or bilateral salpingo-oophorectomy. Table S2. Clinicopathologic characteristics of the training and testing population. Table S3. The selection of bilateral salpingo-oophorectomy and ovarian preservation in the lower or higher risk of death and relapse groups. [file 12957_2023_3014_MOESM5_ESM.docx]

**Table S1.** **Characteristics between women ≤46 years of age with ovarian preservation or bilateral salpingo-oophorectomy.**

| **Variable** | | | | **n=64** | **Ovary saved** | | |  |
| --- | --- | --- | --- | --- | --- | --- | --- | --- |
|  |  |  |  |  | **BSO** | **OP** | **p** |  |
|  |  |  |  |  | **34** | **30** |  |  |
| **FIGO stage (%)** | | | | | | | 0.227 |  |
|  | I | | | 44 (68.8) | 21 (61.8) | 23 (76.7) |  |  |
|  | II | | | 3 (4.7) | 1 (2.9) | 2 (6.7) |  |  |
|  | III | | | 15 (23.4) | 10 (29.4) | 5 (16.7) |  |  |
|  | IV | | | 2 (3.1) | 2 (5.9) | 0 (0.0) |  |  |
| **Tumor size (%)** | | | | | | | 0.044 |  |
|  | ≤2.4cm | | | 22 (38.6) | 7 (24.1) | 15 (53.6) |  |  |
|  | >2.4cm | | | 35 (61.4) | 22 (75.9) | 13 (46.4) |  |  |
| **Preoperative HPV (%)** | | | | | | | 0.755 |  |
|  | negative | | | 3 (7.3) | 1 (4.2) | 2 (11.8) |  |  |
|  | positive | | | 38 (92.7) | 23 (95.8) | 15 (88.2) |  |  |
|  |  | **Positive** | |  |  |  |  | |
|  |  | unknown specific type | | 6 (15.8) | 3 (13.0) | 3 (20.0) |  | |
|  |  | Known | |  |  |  |  | |
|  |  |  | HPV16 | 8 (25.0) | 5 (25.0) | 3 (25.0) |  | |
|  |  |  | HPV18 | 24 (75.0) | 15 (75.0) | 9 (75.0) |  | |
|  |  |  | other 12 high-risk HPVs | 5 (15.6) | 3 (15.0) | 2 (16.7) |  | |
| **Histological heterogeneity (%)** | | | | | | | 0.558 |  |
|  | pure | | | 27 (42.2) | 16 (47.1) | 11 (36.7) |  |  |
|  | mix | | | 37 (57.8) | 18 (52.9) | 19 (63.3) |  |  |
|  |  | **Mix** | | 37 |  |  | 0.447 |  |
|  |  | NEC differentiation | | 7 (18.9) | 2 (11.1) | 5 (26.3) |  |  |
|  |  | NECC dominant | | 30 (81.1) | 16 (88.9) | 14 (73.7) |  |  |
|  |  | **Mix** | | 37 |  |  | 0.858 |  |
|  |  | Squamous | | 6 (16.2) | 3 (16.7) | 3 (15.8) |  |  |
|  |  | Adenocarcinoma | | 28 (75.7) | 14 (77.8) | 14 (73.7) |  |  |
|  |  | both | | 3 (8.1) | 1 (5.6) | 2 (10.5) |  |  |
| **LNM (%)** | | | | | | | 0.405 |  |
|  | negative | | | 47 (73.4) | 23 (67.6) | 24 (80.0) |  |  |
|  | positive | | | 17 (26.6) | 11 (32.4) | 6 (20.0) |  |  |
|  |  | **Positive** | | 17 |  |  | 0.746 |  |
|  |  | pelvic | | 15 (88.2) | 9 (81.8) | 6 (100.0) |  |  |
|  |  | pelvic &para-aortic | | 2 (11.8) | 2 (18.2) | 0 (0.0) |  |  |
|  |  | **Positive** | | 17 |  |  | 0.159 |  |
|  |  | LNM ratio low | | 12 (70.6) | 6 (54.5) | 6 (100.0) |  |  |
|  |  | LNM ratio high | | 5 (29.4) | 5 (45.5) | 0 (0.0) |  |  |
| **Parametrial involvement (%)** | | | | | | | 0.085 |  |
|  | negative | | | 59 (92.2) | 29 (85.3) | 30 (100.0) |  |  |
|  | positive | | | 5 (7.8) | 5 (14.7) | 0 (0.0) |  |  |
| **Vaginal invasion (%)** | | | | | | | 0.056 |  |
|  | negative | | | 54 (85.7) | 26 (76.5) | 28 (96.6) |  |  |
|  | positive | | | 9 (14.3) | 8 (23.5) | 1 (3.4) |  |  |
| **Incisal margin (%)** | | | | | | | 1 |  |
|  | negative | | | 61 (98.4) | 32 (97.0) | 29 (100.0) |  |  |
|  | positive | | | 1 (1.6) | 1 (3.0) | 0 (0.0) |  |  |
| **LUSI (%)** | | | | | | | 0.788 |  |
|  | negative | | | 58 (90.6) | 30 (88.2) | 28 (93.3) |  |  |
|  | positive | | | 6 (9.4) | 4 (11.8) | 2 (6.7) |  |  |
| **DIM (%)** | | | | | | | 0.159 |  |
|  | Superficial 1/3 | | | 21 (33.3) | 9 (27.3) | 12 (40.0) |  |  |
|  | Middle 1/3 | | | 22 (34.9) | 10 (30.3) | 12 (40.0) |  |  |
|  | Deep 1/3 | | | 20 (31.7) | 14 (42.4) | 6 (20.0) |  |  |
| **LVSI (%)** | | | | | | | 1 |  |
|  | negative | | | 10 (16.7) | 6 (18.2) | 4 (14.8) |  |  |
|  | positive | | | 50 (83.3) | 27 (81.8) | 23 (85.2) |  |  |
| **Radiotherapy (%)** | | | | | | | 0.581 |  |
|  | unaccepted | | | 12 (22.6) | 5 (17.9) | 7 (28.0) |  |  |
|  | accepted | | | 41 (77.4) | 23 (82.1) | 18 (72.0) |  |  |

**Abbreviations**

BSO: bilateral salpingo-oophorectomy; OP: ovarian preservation; FIGO: International Federation of Gynecology and Obstetrics; HPV: human papillomavirus; NEC: neuroendocrine carcinoma; NECC: high-grade neuroendocrine cervical carcinoma; LNM: lymph node metastasis; LUSI: lower uterine segment involvement; DIM: depth of myometrial invasion; LVSI: lymph vascular space invasion.

**Table S2. Clinicopathologic characteristics of the training and testing population.**

| **Variable** | | | | **n=116** | **Cohort** | |
| --- | --- | --- | --- | --- | --- | --- |
|  |  |  |  |  | **Training** | **Testing** |
|  |  |  |  |  | **70** | **46** |
| **Age (%)** | | | | | | |
|  | ≤46 | | | 64 (55.2) | 33 (47.1) | 31 (67.4) |
|  | >46 | | | 52 (44.8) | 37 (52.9) | 15 (32.6) |
| **FIGO stage (%)** | | | | | | |
|  | I | | | 71 (61.2) | 43 (61.4) | 28 (60.9) |
|  | II | | | 13 (11.2) | 10 (14.3) | 3 (6.5) |
|  | III | | | 30 (25.9) | 16 (22.9) | 14 (30.4) |
|  | IV | | | 2 (1.7) | 1 (1.4) | 1 (2.2) |
| **Tumor size (%)** | | | | | | |
|  | ≤2.4cm | | | 34 (32.7) | 24 (37.5) | 10 (25.0) |
|  | >2.4cm | | | 70 (67.3) | 40 (62.5) | 30 (75.0) |
| **Preoperative HPV (%)** | | | | | | |
|  | negative | | | 7 (8.9) | 5 (10.2) | 2 (6.7) |
|  | positive | | | 72 (91.1) | 44 (89.8) | 28 (93.3) |
|  |  | **Positive** | |  |  |  |
|  |  | unknown specific type | | 13 (18.1) | 8 (18.2) | 5 (17.9) |
|  |  | Known | |  |  |  |
|  |  |  | HPV16 | 8 (22.2) | 8 (22.2) | 6 (26.1) |
|  |  |  | HPV18 | 29 (80.6) | 29 (80.6) | 17 (73.9) |
|  |  |  | other 12 high-risk HPVs | 6 (16.7) | 6 (16.7) | 4 (17.4) |
| **Ovarian preservation (%)** | | | | | | |
|  | BSO | | | 86 (74.1) | 51 (72.9) | 35 (76.1) |
|  | OP | | | 30 (25.9) | 19 (27.1) | 11 (23.9) |
| **Histological heterogeneity (%)** | | | | | | |
|  | pure | | | 47 (40.5) | 27 (38.6) | 20 (43.5) |
|  | mix | | | 69 (59.5) | 43 (61.4) | 26 (56.5) |
|  |  | **Mix** | | 69 |  |  |
|  |  | NEC differentiation | | 12 (17.4) | 8 (18.6) | 4 (15.4) |
|  |  | NECC dominant | | 57 (82.6) | 35 (81.4) | 22 (84.6) |
|  |  | **Mix** | | 69 |  |  |
|  |  | Squamous | | 12 (17.4) | 7 (16.3) | 5 (19.2) |
|  |  | Adenocarcinoma | | 54 (78.3) | 34 (79.1) | 20 (76.9) |
|  |  | both | | 3 (4.3) | 2 (4.7) | 1 (3.8) |
| **LNM (%)** | | | | | | |
|  | negative | | | 84 (72.4) | 52 (74.3) | 32 (69.6) |
|  | positive | | | 32 (27.6) | 18 (25.7) | 14 (30.4) |
|  |  | **Positive** | | 32 |  |  |
|  |  | pelvic | | 28 (87.5) | 16 (88.9) | 12 (85.7) |
|  |  | pelvic &para-aortic | | 4 (12.5) | 2 (11.1) | 2 (14.3) |
|  |  | **Positive** | | 32 |  |  |
|  |  | LNM ratio low | | 21 (65.6) | 13 (72.2) | 8 (57.1) |
|  |  | LNM ratio high | | 11 (34.4) | 5 (27.8) | 6 (42.9) |
| **Parametrial involvement (%)** | | | | | | |
|  | negative | | | 105 (90.5) | 64 (91.4) | 41 (89.1) |
|  | positive | | | 11 (9.5) | 6 (8.6) | 5 (10.9) |
| **Vaginal invasion (%)** | | | | | | |
|  | negative | | | 91 (79.8) | 55 (79.7) | 36 (80.0) |
|  | positive | | | 23 (20.2) | 14 (20.3) | 9 (20.0) |
| **Incisal margin (%)** | | | | | | |
|  | negative | | | 107 (93.9) | 67 (95.7) | 40 (90.9) |
|  | positive | | | 7 (6.1) | 3 (4.3) | 4 (9.1) |
| **LUSI (%)** | | | | | | |
|  | negative | | | 94 (81.0) | 57 (81.4) | 37 (80.4) |
|  | positive | | | 22 (19.0) | 13 (18.6) | 9 (19.6) |
| **DIM (%)** | | | | | | |
|  | Superficial 1/3 | | | 32 (28.1) | 19 (27.9) | 13 (28.3) |
|  | Middle 1/3 | | | 38 (33.3) | 22 (32.4) | 16 (34.8) |
|  | Deep 1/3 | | | 44 (38.6) | 27 (39.7) | 17 (37.0) |
| **LVSI (%)** | | | | | | |
|  | negative | | | 17 (15.6) | 12 (18.8) | 5 (11.1) |
|  | positive | | | 92 (84.4) | 52 (81.2) | 40 (88.9) |
| **Radiotherapy (%)** | | | | | | |
|  | unaccepted | | | 18 (18.0) | 11 (18.0) | 7 (17.9) |
|  | accepted | | | 82 (82.0) | 50 (82.0) | 32 (82.1) |

**Abbreviations**

FIGO: International Federation of Gynecology and Obstetrics; HPV: human papillomavirus; BSO: bilateral salpingo-oophorectomy; OP: ovarian preservation; NEC: neuroendocrine carcinoma; NECC: high-grade neuroendocrine cervical carcinoma; LNM: lymph node metastasis; LUSI: lower uterine segment involvement; DIM: depth of myometrial invasion; LVSI: lymph vascular space invasion.

**Table S3. The selection of bilateral salpingo-oophorectomy and ovarian preservation in the lower or higher risk of death and relapse groups.**

| **Groups** | |  | **Ovarian preservation** | |
| --- | --- | --- | --- | --- |
|  |  |  | **BSO** | **OP** |
| **Relapse risk (training cohort of DFS)** | | **N=70** | | |
|  | Lower risk | 51 (72.9) | 34 (66.7) | 17 (89.5) |
|  | Higher risk | 19 (27.1) | 17 (33.3) | 2 (10.5) |
| **Relapse risk (testing cohort of DFS)** | | **N=46** | | |
|  | Lower risk | 36 (78.3) | 25 (71.4) | 11 (100.0) |
|  | Higher risk | 10 (21.7) | 10 (28.6) | 0 (0.0) |
| **Death risk (training cohort of OS)** | | **N=70** | | |
|  | Lower risk | 49 (70.0) | 31 (60.8) | 18 (94.7) |
|  | Higher risk | 21 (30.0) | 20 (39.2) | 1 (5.3) |
| **Death risk (testing cohort of OS)** | | **N=46** | | |
|  | Lower risk | 33 (71.7) | 23 (65.7) | 10 (90.9) |
|  | Higher risk | 13 (28.3) | 12 (34.3) | 1 (9.1) |
| **Relapse risk (≤46 years old)** | | **N=64** | | |
|  | Lower risk | 28 (43.8) | 12 (35.3) | 16 (53.3) |
|  | Higher risk | 36 (56.2) | 22 (64.7) | 14 (46.7) |

**Abbreviations**

BSO: bilateral salpingo-oophorectomy; OP: ovarian preservation; OS: overall survival; DFS: disease-free survival.
