# Supplementary material for: Impacts of ovarian preservation on the prognosis of neuroendocrine cervical carcinoma: a retrospective analysis based on machine learning
Source: World J Surg Oncol. 2023 May 12;21:146. doi: 10.1186/s12957-023-03014-9 (PMC10176922; doi:10.1186/s12957-023-03014-9)
Supplement: Supplementary file 9 — Additional file 9: Table S7. Clinical and pathological characteristics of patients with ovarian metastasis. [file 12957_2023_3014_MOESM9_ESM.docx]

**Table S7. Clinical and pathological characteristics of patients with ovarian metastasis.**

| **Patient** | 1 | 2 | 3 |
| --- | --- | --- | --- |
| **Age** | 61 | 53 | 30 |
| **Stage (FIGO, 2018)** | IIA1 | IIIC2p | IVA |
| **Tumor diameter (cm)** | NA | 5 | NA |
| **Pathological diagnosis** | NECC and invasive adenocarcinoma | NECC | SCNECC |
| **LNM** | Negative | Positive (pelvic and para-aortic lymph nodes involved, positive ratio 27.27%) | Positive (pelvic and para-aortic lymph nodes involved, positive ratio 96.00%) |
| **Parametrial involvement** | Negative | Bilateral involved | Bilateral involved |
| **Vaginal invasion** | Positive | Positive | Negative |
| **Incisal margin** | Positive | Negative | NA |
| **LUSI** | Negative | Positive | Positive |
| **DIM** | Middle 1/3 | Deep 1/3 | Deep 1/3 |
| **LVSI** | Positive (CD31 and D240) | Positive (extensively) | Positive (extensively, CD31 and D240) |

**Abbreviations**

FIGO: International Federation of Gynecology and Obstetrics; SCNECC: small cell neuroendocrine cervical carcinoma; NECC: high-grade neuroendocrine cervical carcinoma; LNM: lymph node metastasis; LUSI: lower uterine segment involvement; DIM: depth of myometrial invasion; LVSI: lymph vascular space invasion.
