# Supplementary material for: Impacts of ovarian preservation on the prognosis of neuroendocrine cervical carcinoma: a retrospective analysis based on machine learning
Source: World J Surg Oncol. 2023 May 12;21:146. doi: 10.1186/s12957-023-03014-9 (PMC10176922; doi:10.1186/s12957-023-03014-9)
Supplement: Supplementary file 3 — Additional file 3: Supplementary Fig. 3. (A) LASSO coefficient profiles of the 11 key clinicopathological variables for the prediction of DFS in the training cohort. (B) Tuning parameter selection by tenfold cross-validation in the LASSO model of training cohort. The partial likelihood deviance was plotted against log (Lambda/λ), and λ was the tuning parameter. The partial likelihood deviance values were shown and error bars represented s.e. The dotted vertical lines showed the optimal values through minimum criteria and 1-s.e. criteria. (C) Tenfold cross-validation showed that 4 variables could minimize the error of optimum subsets regression analysis in the training cohort. The “bestglm” R package was used. (D) The Cox model display the variables related to DFS chosen by stepwise regression in the training cohort. (E) LASSO coefficient profiles of the 13 key clinicopathological variables for the prediction of OS in the training cohort. (F) Tuning parameter selection by tenfold cross-validation in the LASSO model of training cohort. (G) Tenfold cross-validation showed that 3 variables could minimize the error of optimum subsets regression analysis in the training cohort. (H) The Cox model of the variables related to OS chosen by stepwise regression in the training cohort. (I-J) The proportion of relapse in high and low risk groups of the training and testing cohort as risk scores increased. (K-M) ROC of the random forest model for relapse at 1, 3, and 5 years in the training, testing cohort, and all patients. (N) KM curves of DFS between lower and higher risk groups classified by random forest analysis in all patients. (O-P) KM curves of DFS between BSO and OP groups in low DFS risk patients of training and testing cohort. (Q-R) The proportion of death in high and low risk groups of the training and testing cohort as risk scores increased. (S-U) ROC of the random forest model for death at 1, 3, and 5 years in the training, testing cohort, and all patients. (V) KM c [file 12957_2023_3014_MOESM3_ESM.pdf]

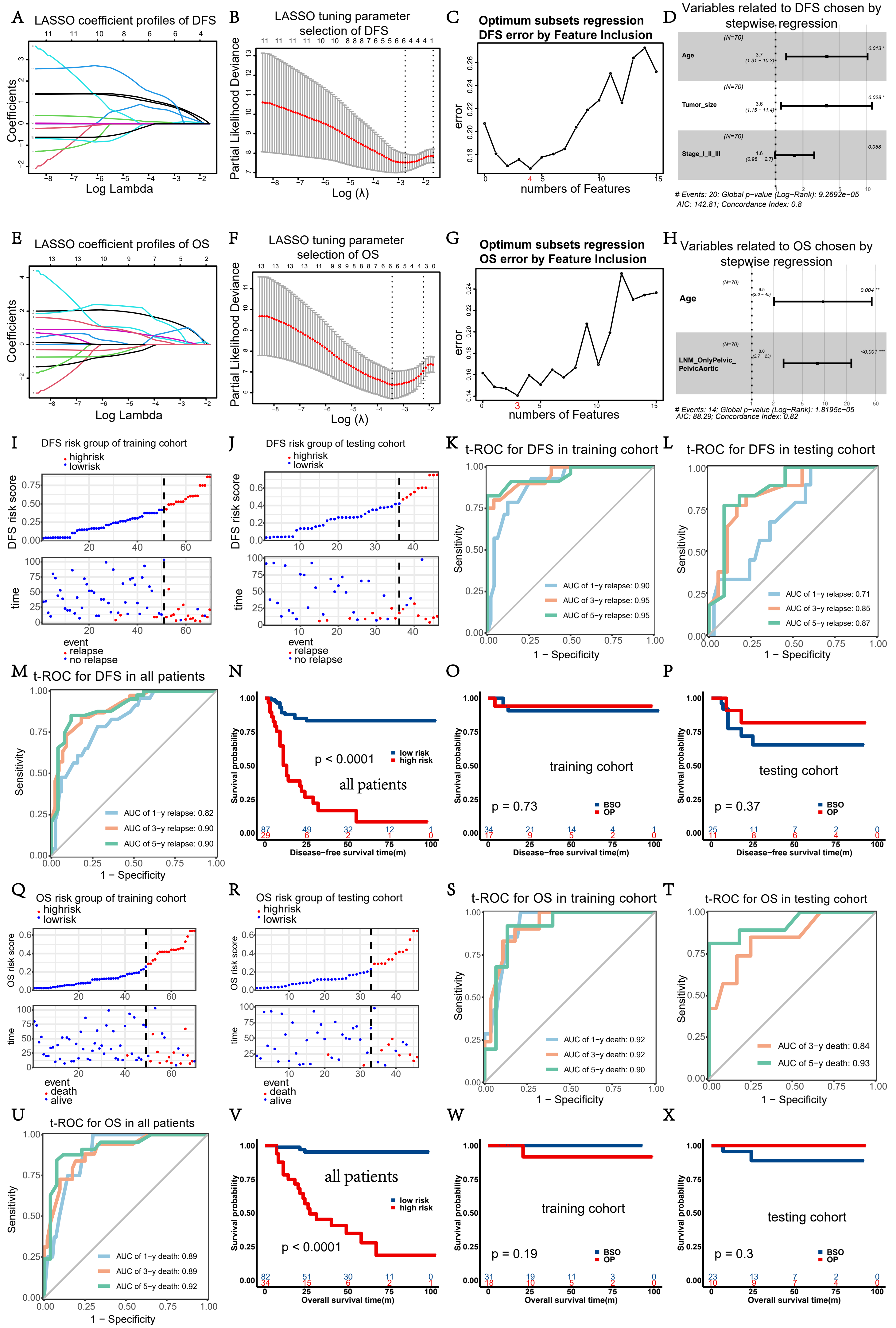
A LASSO coefficient profiles of DFS
Coefficients
Log Lambda
B LASSO tuning parameter selection of DFS
Partial Likelihood Deviance
Log (λ)
C Optimum subsets regression DFS error by Feature Inclusion
error
numbers of Features
D Variables related to DFS chosen by stepwise regression
Age (N=70) 3.7 (1.31 − 10.3) 0.013 *
Tumor_size (N=70) 3.6 (1.15 − 11.4) 0.028 *
Stage_I_II_III (N=70) 1.6 (0.98 − 2.7) 0.058
Events: 20; Global p-value (Log-Rank): 9.2692e−05
AIC: 142.81; Concordance Index: 0.8
E LASSO coefficient profiles of OS
Coefficients
Log Lambda
F LASSO tuning parameter selection of OS
Partial Likelihood Deviance
Log (λ)
G Optimum subsets regression OS error by Feature Inclusion
error
numbers of Features
H Variables related to OS chosen by stepwise regression
Age (N=70) 9.5 (2.0 − 45) 0.004 **
LNM_OnlyPelvic_PelvicAortic (N=70) 8.0 (2.7 − 23) <0.001 ***
Events: 14; Global p-value (Log-Rank): 1.8195e−05
AIC: 88.29; Concordance Index: 0.82
I DFS risk group of training cohort
highrisk
lowrisk
DFS risk score
time
event
relapse
no relapse
J DFS risk group of testing cohort
highrisk
lowrisk
DFS risk score
time
event
relapse
no relapse
K t-ROC for DFS in training cohort
Sensitivity
1 − Specificity
AUC of 1−y relapse: 0.90
AUC of 3−y relapse: 0.95
AUC of 5−y relapse: 0.95
L t-ROC for DFS in testing cohort
AUC of 1−y relapse: 0.71
AUC of 3−y relapse: 0.85
AUC of 5−y relapse: 0.87
M t-ROC for DFS in all patients
AUC of 1−y relapse: 0.82
AUC of 3−y relapse: 0.90
AUC of 5−y relapse: 0.90
N Survival probability
p < 0.0001
all patients
low risk
high risk
Disease−free survival time(m)
O training cohort
p = 0.73
BSO
OP
Disease−free survival time(m)
P testing cohort
p = 0.37
BSO
OP
Disease−free survival time(m)
Q OS risk group of training cohort
highrisk
lowrisk
OS risk score
time
event
death
alive
R OS risk group of testing cohort
highrisk
lowrisk
OS risk score
time
event
death
alive
S t-ROC for OS in training cohort
AUC of 1−y death: 0.92
AUC of 3−y death: 0.92
AUC of 5−y death: 0.90
T t-ROC for OS in testing cohort
AUC of 3−y death: 0.84
AUC of 5−y death: 0.93
U t-ROC for OS in all patients
AUC of 1−y death: 0.89
AUC of 3−y death: 0.89
AUC of 5−y death: 0.92
V all patients
low risk
high risk
p < 0.0001
Overall survival time(m)
W training cohort
p = 0.19
BSO
OP
Overall survival time(m)
X testing cohort
p = 0.3
BSO
OP
Overall survival time(m)
